# kMetaShot: a fast and reliable taxonomy classifier for metagenome-assembled genomes

**DOI:** 10.1093/bib/bbae680

**Published:** 2025-01-02

**Authors:** Giuseppe Defazio, Marco Antonio Tangaro, Graziano Pesole, Bruno Fosso

**Affiliations:** Department of Biosciences, Biotechnology and Environment, University of Bari Aldo Moro, Via E. Orabona 4, 70126, Bari, Italy; Institute of Biomembranes, Bioenergetics and Molecular Biotechnologies, Consiglio Nazionale delle Ricerche, Via G. Amendola 122/O, 70125, Bari, Italy; Department of Biosciences, Biotechnology and Environment, University of Bari Aldo Moro, Via E. Orabona 4, 70126, Bari, Italy; Institute of Biomembranes, Bioenergetics and Molecular Biotechnologies, Consiglio Nazionale delle Ricerche, Via G. Amendola 122/O, 70125, Bari, Italy; Consorzio Interuniversitario Biotecnologie, BIC Incubatori, Via Flavia 23/1, 34148, Trieste, Italy; Department of Biosciences, Biotechnology and Environment, University of Bari Aldo Moro, Via E. Orabona 4, 70126, Bari, Italy

**Keywords:** shotgun metagenomics, taxonomic classification, *k*-mer, minimizer

## Abstract

The advent of high-throughput sequencing (HTS) technologies unlocked the complexity of the microbial world through the development of metagenomics, which now provides an unprecedented and comprehensive overview of its taxonomic and functional contribution in a huge variety of macro- and micro-ecosystems. In particular, shotgun metagenomics allows the reconstruction of microbial genomes, through the assembly of reads into MAGs (metagenome-assembled genomes). In fact, MAGs represent an information-rich proxy for inferring the taxonomic composition and the functional contribution of microbiomes, even if the relevant analytical approaches are not trivial and still improvable. In this regard, tools like CAMITAX and GTDBtk have implemented complex approaches, relying on marker gene identification and sequence alignments, requiring a large processing time. With the aim of deploying an effective tool for fast and reliable MAG taxonomic classification, we present here kMetaShot, a taxonomy classifier based on *k*-mer/minimizer counting. We benchmarked kMetaShot against CAMITAX and GTDBtk by using both *in silico* and real mock communities and demonstrated how, while implementing a fast and concise algorithm, it outperforms the other tools in terms of classification accuracy. Additionally, kMetaShot is an easy-to-install and easy-to-use bioinformatic tool that is also suitable for researchers with few command-line skills. It is available and documented at https://github.com/gdefazio/kMetaShot.

## Introduction

Microbial ecology, namely, the investigation of microbes, their mutual interactions, and their environmental impact [1], has been progressively enhanced by the advent of new technologies, such as microscopy, culture-based approaches, and sequencing [2]. Since the association between infectious disease and microbe infections was established, a paradigm on the identification and eradication of pathogens was also established [3]. A deeper understanding of the microbial world highlighted pathogens representing only a tiny part of the whole microscopic complexity [2], and we learned that microorganisms can live everywhere and tend to aggregate in complex communities [4, 5]. Through the years, approaches relying on bacteria cultivation enabled the isolation and characterization of several bacterial species resulting in an operative definition of prokaryotic species, based on DNA–DNA hybridization (DDH). Conspecific strains should show at least 70% DDH, not only implying genomic cohesion (a measure of within-species similarity) but also suggesting a wide phenotypic variability [6, 7]. More recently, the advent of high-throughput sequencing (HTS) technologies produced a big leap forward in our understanding of microbes, allowing us to access complete genomes and their functional potentials [8], resulting in a revision of species definition, based on the comparison of average nucleotide similarity index (ANI) [9, 10]. Culture-free approaches, and in particular shotgun metagenomics and DNA metabarcoding [11–13], allowed a more exhaustive understanding of microbial communities and their crucial role for a large variety of ecosystems [14–16] including different host districts in humans (e.g. gut), animals, and plants [17]. HTS data, although initially used for the taxonomic characterization at read level [18–24], may allow the reconstruction of nearly complete genomes, denoted as MAGs (metagenome-assembled genomes), through bioinformatics approaches for assembly and binning [25] and also be able to assess their gene and metabolic potential. MAGs do not fully recapitulate the features of genomes obtained from isolates and pure cultures (i.e. type strains) that are usually ungapped circular sequences [26]. Nonetheless, they remarkably allow us to obtain accurate taxonomic information to identify new taxa and their ecological role [27]. Despite the fact that MAGs are useful for representing genomes in a community, they are prone to some intrinsic limitations. MAGs cannot discriminate among near-clonal subpopulations and so are considered ‘population consensus’ genomes and chimeric [8, 28]. A possible alternative to MAGs is represented by single amplified genomes (SAGs), in which single-cell applications are used to retrieve genomes from microbial cells. Nonetheless, SAGs are characterized by higher costs and low throughput and quality, the latter principally due to underamplification that leads to loss of genomic content [8, 29].

Although the characterization of single genomes from complex communities remains a crucial goal, it is also fundamental to better understand its overall functional contribution. A microbiome is made up of living microorganisms (actually the microbiota) and their theatre of activities [30]. The metagenome corresponds to the genomic and genetic content of the microbiome. The exchange of metabolites and molecular signals allows microbes to detect and get in touch with other members of the community [31], resulting in a fine suborganization of the community itself based on a microbial guild specialized in addressing specific tasks and bioprocesses [32]. Consequently, focusing on prokaryotes becomes crucial to profile the community at the strain level (or at least at the subspecies level) to properly understand the range of possible interactions between microbes and the environment they colonize, regardless of whether it is the human gut or soil [12, 33, 34]. Currently, MAG-based approaches represent an effective viable way to unveil microbial genetic complexity and can be obtained by binning meta-assembled HTS data (i.e. contigs) according to sequence features (e.g. tetranucleotide frequency, GC%) [35, 36]. MAG taxonomic classification is an expanding field where few bioinformatic tools are effectively used in the community, and GTDBtk [37, 38] and CAMITAX [39], can be considered the gold standards. They rely on alignment-based and marker gene identification approaches, performing the classification up to the species level. Remarkably, GTDBtk was used, for example, in the MAG taxonomic classification of infants’ gut microbiota and evaluate antibiotic exposure impact [40], while CAMITAX was used in the second Critical Assessment of Metagenome Interpretation (CAMI) challenge [41]. In general, both GTDBtk and CAMITAX are workflows wrapping different tools, and although the combination of several approaches provides quite reliable classifications, the demand for computational resources is huge.

In consideration of the ever-increasing amount of data produced by HTS technologies and the need to optimize their information mining, approaches based on *k*-mer (i.e. contiguous nucleotide strings of fixed length *k*) counting have been largely used for different applications [42], such as genome/metagenome assembly [43–47], read classification [19, 20, 48], and mapping [49]. Considering that *k*-mers and minimizers (i.e. *k*-mers representing a group of *k*-mers [42]) have been already successfully used to extrapolate specific genomic signatures from large datasets [50], we developed kMetaShot, a bioinformatic approach relying on *k*-mer/minimizer profiling from the reference prokaryotic genomes, in order to build a concise representation of genomic diversity and perform MAG taxonomic classification up to the strain level. In this work, we demonstrate its superior accuracy in taxonomic classification by performing extensive benchmarking against GTDBtk and CAMITAX. Moreover, considering the current effort to improve the computational efficiency of bioinformatic approaches, in terms of storage and Central Processing Unit (CPU) time, we also demonstrated that kMetaShot is computationally less expensive compared to the benchmarked tools, with very user-friendly installation and usage procedures. In conclusion, we believe that kMetaShot represents a tangible advance at both scientific and computational levels by providing reliable characterization of microbial communities up to the subspecies level and reducing requests in computational resources.

## Materials and methods

**Figure 1:**
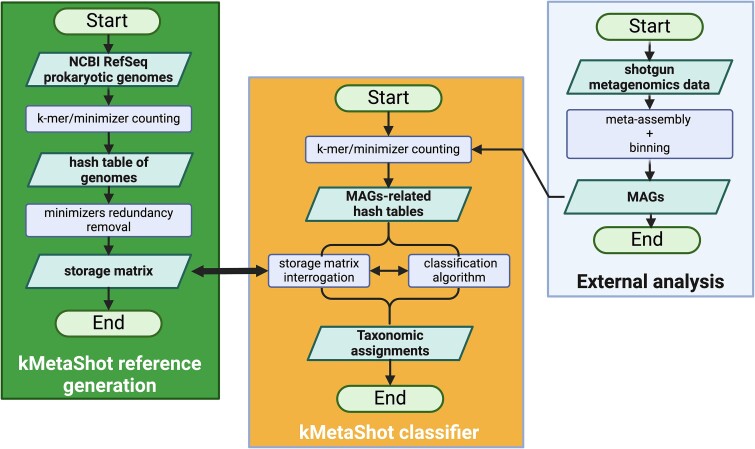
Flowchart representing the kMetaShot workflow. In particular, left (green) and central (yellow) boxes refer to reference generation and classification modules, respectively. Both modules rely on *k*-mer/minimizer counting. The reference generation module aims to identify and store nonredundant minimizers in the storage matrix. The classification module compares the minimizer found in MAGs to those in the storage matrix. Finally, the right (light-blue) box refers to a general workflow for MAG generation.

kMetaShot is based on the reference and the classifier modules (Fig. 1). The reference module is designed to collect relevant minimizers at ‘genus’ and ‘strain’ levels from bacterial and archaeal genomes. The classifier module provides MAG taxonomy by comparing their minimizers’ profiles to a reference.

kMetaShot has been initially tested on HMP (Human Microbiome Project) [51] genomes for tuning and then benchmarked against GTDBtk and CAMITAX using several *in silico* and real mock communities.

### kMetaShot reference module

CDSs (coding sequences) and ncRNAs (noncoding RNAs) from RefSeq Bacterial and Archaeal genomes (Release 213) [52] were *k*-mer- and minimizer-counted to define a set of nonredundant minimizers for each genome. Then, relying on the National Center for Biotechnology Information (NCBI) taxonomy, the algorithm focused on genus and strain levels to discard ambiguous minimizers (i.e. those shared among genomes belonging to different ‘genera’) and retain those exclusively represented in genome(s) of the same ‘strain’ or ‘genus’. Retained minimizers and related taxonomy identifiers (taxids) are stored in a matrix.

The first step of kMetaShot reference generation consisted of Archaea and Eubacteria RefSeq CDS (i.e. coding sequences) and ncRNA (i.e. ribosomal, transfer) sequence data retrieval. This approach allowed us to reduce data elaborations compared to consider the whole genome. To simplify the description, hereinafter, the CDS and ncRNA collections obtained are referred to as genomes, taking into account that prokaryotic coding sequences represent at least 80% of the entire bacterial genome [53]. Concurrently, the NCBI Taxonomy and the assembly summary files, which contain all the relevant features of the RefSeq genomes, were also downloaded. Indeed, the assembly summary file contains the ‘taxid’ column storing the lowest taxonomic classification associated with each genome, according to NCBI Taxonomy, and allowing us to infer the taxonomic path. Only eight taxonomic ranks (i.e. superkingdom, phylum, class, order, family, genus, species, strain) were considered. The kMetaShot reference and taxonomy classification system relies exclusively on ‘genus’ and ‘strain’ ranks, and particular attention was paid to the refinement of these two ranks. Initially, it compared the identifiers stored in the ‘taxid’ and ‘species’ assembly summary fields. If different, kMetaShot assumed the available identifier in the taxid column as strain. It is crucial to note that for the aim of this paper, we consider the strain as a descriptive subdivision of a species [8]. Otherwise, the kMetaShot reference generator applied a strain refinement procedure. Therefore, for each set of genomes belonging to the same species and lacking strain classification, the similarity between genomic sequences was assessed using Mash [54] to quickly estimate the average nucleotide identity (ANI). Then, squared matrices collecting pairwise ANI values between genomes are used to perform clustering, using DBSCAN (Density-Based Spatial Clustering of Applications with Noise) with cluster similarity settled at 97%. Although the strain clustering threshold was arbitrary, it took into account that the operational ANI threshold to define a species is 95% [55]. Before clustering, genomes that obtained an ANI <50% were considered outliers and removed from subsequent analysis. After clustering, each cluster represented a ‘convenience strain’, an expedient of kMetaShot to standardize its reference taxonomy, that is, to obtain a comparable length for all taxonomic paths of genomes. A new taxonomic identifier is assigned to each cluster, corresponding to integers in the interval ranging between the highest used number as the taxonomic identifier in the NCBI taxonomy and 2^32^-1. Finally, genomes lacking a taxonomic classification at the genus level are labelled using the lowest taxonomy identifier in the superkingdom-family interval as an ‘operative’ genus identifier. The careful curation of taxonomic information is necessary to obtain a reliable system capable of retaining sequence features and correctly using them for optimizing the accuracy of taxonomic assignment.

The genomes were then processed through *k*-mer/minimizer count to generate the related ‘hash tables’. Overall, a *k*-mer-counting procedure relies on the decomposition of sequences into substrings of fixed length *k*, the so-called *k*-mers. The *k*-mer-counting procedure could ideally be resumed by recording a sliding window that moves through the sequence one nucleotide per time. The result of this procedure is a table containing the observed *k*-mers and their occurrences, inferred both on a sequence and its reverse complement. Nevertheless, *k*-mer counting is not sufficient to achieve a concise genome representation and fast data querying. For this reason, a minimizer system has been implemented in kMetaShot.

**Figure 2:**
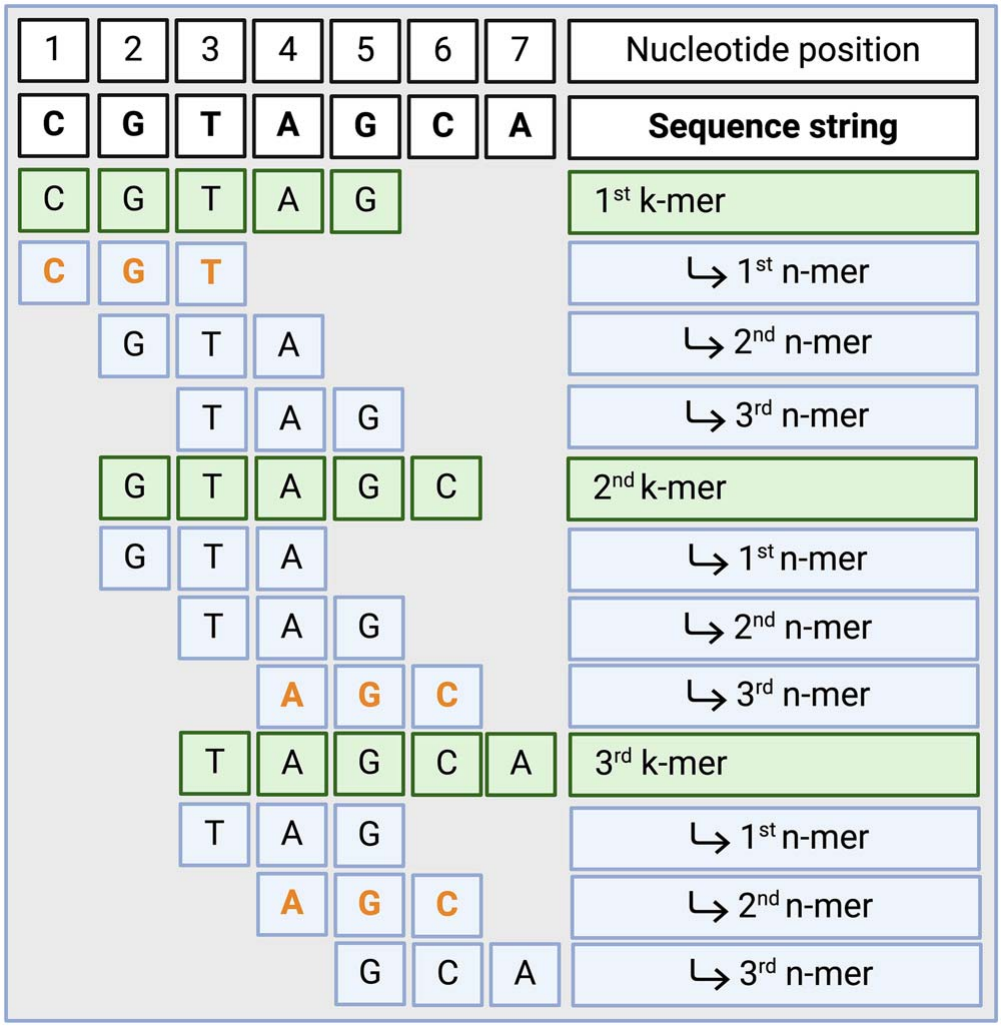
Example of minimizer computation. The nucleotide sequence is firstly *k*-mer-counted. Each *k*-mer is decomposed in *n*-mers, and the *n*-mer that minimizes the lexicographical order is selected as a minimizer. The lexicographical order is A < C < G < T. Selected minimizer sequences are coloured in orange. *k* = 5, *n* = 3.

Briefly, if the *k*-mer is a DNA substring *k* nucleotides long, a minimizer is a *k*-mer substring *n* nucleotides long, chosen using a minimizing criterion to synthetically represent a *k*-mer [56]. The minimizer application was aimed at compressing the information to store. Overall, following the choice of a minimizing criterion (i.e. lexicographical order, arbitrary scoring table, etc.), every single *k*-mer is further decomposed into *n*-mers, with *k* > *n*. Then, for each *k*-mer, the *n*-mer that minimizes the chosen criterion is used for concise *k*-mer representation (Fig. 2). Actually, if two sequences are similar, at least one minimizer chosen from the first sequence will be found in the second one [56].

In the kMetaShot *k*-mer/minimizer counting procedure, the lexicographical minimization criterion was used (i.e. A < C < G < T). To address *k*-mer/minimizer counting in sequences containing uncalled bases N or other non-A-T-C-G IUPAC symbols, we initially split them in correspondence with these unconventional nucleotides. Sequence fragments shorter than the imposed *k*-mer length were then dropped. The remaining sequences were *k*-mer/minimizer-counted. The fixed lengths adopted for the *k*-mer and minimizer were 61 and 31, respectively. Textual *k*-mers and minimizers were also binary-compressed by using a 2-bit encode for each nucleotide (Table 1).

**Table 1:** Employed binary conversion table for nucleotides.

**Nucleotide**	**Binary representation**
A	00
C	01
G	10
T	11

Each selected minimizer was represented by a bit array composed of 62 (31 × 2) bits. In order to represent it in an 8-byte array (i.e. 64 bits), the ‘00’ offset was added at position 0 and 1 of the array (i.e. less significant bit positions). Then, the converted 8-byte minimizer was hashed by ‘murmur3’ hashing function [57], resulting in an unsigned integer weighting 32 bits (i.e. 4 bytes). At the end of the process, each minimizer corresponds to a 32-bit unsigned integer. The *k*-mer/minimizer counting procedure was separately applied to each genome. For each genome, a dataset collecting unsigned 32-bit integers corresponding to the counted minimizers was stored.

The kMetaShot reference basically relies on the individuation of relevant minimizer sets at strain and genus levels. Minimizers in common to different genera were flagged as redundant and discarded. Minimizers exclusively shared between genomes belonging to the same strain or genus were considered ‘relevant’ and retained in the storage matrix.

The storage matrix consists of 2^32^ slots distributed in $\frac{2^{32}}{2^3}$ = 2^29^ rows and 8 columns, collecting all the possible minimizers. The slot position and content represent the minimizer and related taxonomic identifier (an unsigned 32-bit integer), respectively. The slot position for each minimizer was computed by converting the numerical value representing a minimizer into a tuple containing row and column matrix coordinates using the following formulas:


$$\kern-1.4pc \text{Row}=\text{minimizer}\div 8 $$

$$ \text{Column}=\text{module}\left(\text{minimizer},8\right) $$

**Figure 3:**
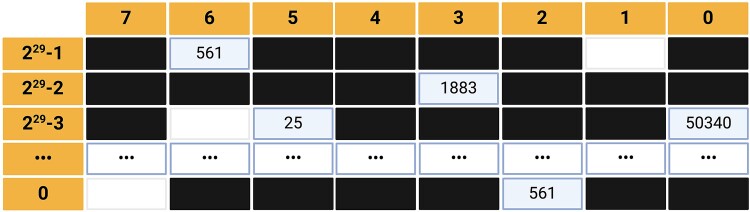
Graphical representation of the storage matrix. Black and white slots represent redundant minimizers (i.e. those shared among different genera) and empty ones, respectively. Filled slots refer to the relevant minimizer. For instance, the slot identified by row 2^29^-2, and Column 3 is associated with taxid 1883 (*Streptomyces*).

In order to find relevant minimizers, the following algorithm was applied:

1. For each strain, the algorithm began by selecting a genome containing a number of minimizers equal to the median values observed for that specific strain; for each minimizer from the selected genome, the algorithm found the related slot position and verified the slot content.
2. If the slot was empty, it filled the slot with the strain taxonomy ID.
3. If the slot was already filled with the same strain or genus identifier, the algorithm considers the minimizer as relevant and the slot remains accessible.
4. Otherwise, if the slot contains a strain identifier belonging to a different genus, the algorithm considers the minimizer redundant and blacks the slot making it no longer accessible (Fig. 3).

At the end of this procedure, the storage matrix represents the whole kMetaShot reference and stores four possible types of information:

- Empty slots for minimizer never observed in the reference genomes (i.e. nullomers [42]);
- Black slot, for redundant minimizers;
- Strain taxonomy identifier for minimizers observed exclusively in a strain;
- Genus taxonomy identifier for genera-associated minimizers.

The weight of the storage matrix was easily estimable by multiplying the weight of a single matrix slot by the number of slots:


$$\kern-4.1pc \text{Slot}=32\ \text{bits}=4\ \text{bytes} $$

$$\kern-9.2pc \text{Nr slots}=2^{32}$$

$$\text{Weight}\ \text{matrix}=4\times 2^{32}=17\ 179\ 869\ 184\ \text{bytes}=\ \sim17\text{Gb}$$

### Taxonomic classification algorithm

The kMetaShot classifier module processes MAGs by shredding query data in minimizer sets and then queries the storage matrix to retrieve related taxonomic information if it is available. Finally, a taxonomic classification is obtained by applying a prevalence criterion. kMetaShot input consists in the MAGs folder embedding binned contigs in FASTA format.

The *k*-mer/minimizer counting on query sequences relies on the same principles, rules, and implementations described for reference building, in order to obtain a set of unsigned 32-bit integers representing minimizers (referred to as query-minimizer, q-minimizer).

In the storage matrix query step, for each q-minimizer, kMetaShot extracts the data stored in the corresponding slot. If the slots are not empty or blacked, it stores the related strain or genus identifier. Then, for each analysed MAG, a resume table collecting the observed taxonomic identifiers, occurrences, and ranks (i.e. strain or genus) is generated.

Finally, classification is achieved using a prevalence-based approach. First, the algorithm assigns the query sequence to the most abundant genus in the resume table and then the species to the most abundant species of the assigned genus. In the last step, the algorithm considers the number of minimizers associated with each observed strain belonging to an assigned species and computes a ratio called ‘ass2ref’ corresponding to $\frac{\text{strain}\ \text{q}-\text{minimizers}}{\text{strain}\ \text{reference}\ \text{minimizers}}$ and assigns the query sequence to the strain maximizing ass2ref. Also, the ass2ref parameter (i.e. ranging between 0 and 1) was implemented as an optional filter to drop out unreliable classifications at strain and species levels, retaining those at the genus level.

The kMetaShot output consists of a CSV (comma-separated value) file containing a line per each classified MAG and 11 fields, namely: (i) bin name; (ii) ass2ref (measured ass2ref); (iii) taxid (kMetaShot Taxonomy Identifier); (iv–x) taxonomic identifier from species to kingdom; and (xi) assigned microorganism name.

### kMetaShot testing on Human Microbiome Project genomes

Initially, kMetaShot was tested on Bacterial and Archaeal HMP (Human Microbiome Project) [51] genomes, accessed on 6 January 2022 at https://ftp.ncbi.nlm.nih.gov/genomes/HUMAN_MICROBIOM/Bacteria/, allowing us to measure the assignment reliability. Taxonomic data for each genome were extracted from the associated flat file. The 939 HMP genomes, related to 934 strains, 556 species, and 190 genera, were downloaded*.* Each genome was taxonomically classified by kMetaShot at the strain, species, and genus level, and the classification accuracy was assessed. Assignment accuracy was evaluated by measuring sensitivity, precision, and the false-positive rate (FPR). More thoroughly, we define *C*_O_ and *C*_E_ as the set of observed and expected classifications, respectively. Finally, *C*_T_ corresponds to all taxa available in the kMetaShot reference taxonomy. Those were used to measure the following metrics:

-
$\text{True}\ \text{positive}\ \left(\text{Tp}\right): C_{O}\cap C_{E}$

-
$\text{False}\ \text{positive}\ \left(\text{Fp}\right): C_{O}\hbox{ - } \left( C_{O}\cap C_{E}\right)$

-
$\text{False}\ \text{negative}\ \left(\text{Fn}\right): C_{E}\hbox{ -} \left( C_{O}\cap C_{E}\right)$

-
$\text{True}\ \text{negative}\ \left(\text{Tn}\right): C_{T}\hbox{ - } \left( C_{O}\cap C{E}\right)$

-
$\text{Sensitivity}\ \left(\text{True}\ \text{Positive}\ \text{Rate}\right):\frac{\text{Tp}}{\text{Tp} \ + \ \text{Fn}}\times 100$

-
$\text{Precision}:\frac{\text{Tp}}{\text{Tp} \ + \ \text{Fp}}\times 100$

-
$\text{False}\ \text{Positive}\ \text{Rate}:\frac{\text{Fp}}{\text{Fp} \ + \ \text{Tn}}$

-
$\text{Balanced}\ \text{accuracy}:\frac{\text{Sensitivity}\ +\ \text{Specificity}}{2}=\frac{\frac{\text{Tp}}{\text{Tp}\ + \ \text{Fn}} + \frac{\text{Tn}}{\text{Tn}\ + \ \text{Fp}}}{2}$

-
$\text{F}1\ \text{score}:\frac{2 \ \times \ \text{Precision} \ \times \ \text{Sensitivity}}{\text{Precision} \ + \ \text{Sensitivity}}$

The results obtained were used to assess the impact of false positives and reduce them by implementing the ass2ref score. This means that instead of performing strain classification using the same prevalence-based approach used for genera and species, we imposed a parameter weighting the observed q-minimizer out of the expected ones. This parameter was also intended as a threshold that users can apply to further filter the results.

Moreover, two state-of-art tools, namely, GTDBtk (v1.0.2) and CAMITAX (v0.7.0; Nextflow v21.04.0) [37, 39], were used to classify HMP genomes, and classification performances were compared to kMetaShot by using sensitivity, precision, FPR, balanced accuracy (BA), and F1-score. Both GTDBtk and CAMITAX did not perform classification at the strain level.

### kMetaShot benchmarking on Critical Assessment of Metagenome Interpretation datasets

kMetaShot was also benchmarked against GTDBtk (v1.0.2) and CAMITAX (v0.7.0; Nextflow v21.04.0) [37, 39] by using *in silico* CAMI II multi-sample mock community datasets [41].

We took advantage of the data produced with the Critical Assessment of Metagenome Interpretation (CAMI) initiative by using three *in silico* produced mock communities to further benchmark kMetaShot. We selected Gastro-Intestinal (GI) and Airways (Air) human-associated samples and plant-associated samples. For GI and Air, CAMI II provided contigs generated by assembling both Illumina paired-end and PacBio simulated reads. Ten samples for each condition (i.e. GI-Illumina, GI-Pacbio, Air-Illumina, Air-PacBio) were randomly extracted and analysed, for a total of 40 samples. Furthermore, 20 plant-associated (PA) datasets containing contigs obtained from simulated PacBio reads (i.e. PA-PacBio) were analysed. For each sample, the provided contigs were binned separately with MetaBAT2 (v2:2.15) [35] (--maxP 99 --minS 98 -m 1,500) to obtain MAGs. Inferred MAGs were taxonomically classified by using kMetaShot, GTDBtk, and CAMITAX. The obtained taxonomic labels were compared with the expected ones, and sensitivity, precision, FPR, BA, and F1-score were measures.

### kMetaShot benchmarking on a real dataset

Furthermore, kMetaShot was benchmarked on real sequencing data from a mock community composed of 48 bacterial and 16 archaeal genomes (Supplementary Table 1). The mock community was sequenced by using the Illumina HiSeq 2000 platform with a paired-end (PE) 2 × 100bp layout (data available on SRA repository: SRR606249). After a quality check with FastQC [58] and trimming with Sickle [59] (-q 32 -l 50), PE reads were meta-assembled with metaSpades (v3.15.2) [44] and MegaHIT (v1.2.9) [45] (for both -k 35, 57, 79, 99). The obtained contigs were binned separately with MetaBAT2 (v2:2.15) [35] (--maxP 99 --minS 98 -m 1,500) to obtain MAGs. The MAG inference was performed by using two different meta-assemblers to assess their impact on classification. kMetaShot, GTDBtk, and CAMITAX were used to taxonomically classify the inferred MAGs.

Actually, 47 out 64 mock genomes held only a species label but not a strain annotation [60]. With MAGs achieving in kMetaShot a strain classification for those species lacking this information, we further performed an ANI comparison, in which these MAGs were compared against all the RefSeq genomes available for the observed strains by exploiting Mash [54]. Squared matrix results were graphically rendered as heat maps. Unlabelled genomes were then assigned to strain with the highest ANI (at least ≥97%). Sensitivity, precision, FDR, balanced accuracy, and F1-score were measured for all the obtained classifications and only for kMetaShot at the strain level were evaluated twice, before and after strain refinement.

### GTDBtk taxonomy conversion

The NCBI taxonomy was employed in HMP genomes, CAMI, and real mock taxa names. kMetaShot and CAMITAX classifications were all based on the NCBI Taxonomy, and no conversion was required. The GTDBtk results were converted from the Genome Taxonomy Database (GTDB) taxonomy to NCBI by using the ncbi_vs_gtdb_r89 conversion tables (available at https://data.gtdb.ecogenomic.org/releases/release89/89.0/).

### Evaluation of computational requirements

Finally, by using the CMDbench Python package [61], benchmarked tools were also evaluated in terms of required memory Random Access Memory (RAM) and CPU% and overall execution time. The datasets with the lowest and largest number of MAGs were employed. In particular, CAMITAX is run through Nextflow, which, by default, exploits Docker containers to parallelize, as much as possible, the analysis steps. This leads to heavily underestimating its CPU and memory consumption with standard tools, which just tracks the main process, Nextflow, without its children processes. Therefore, to estimate the real CPU and memory consumption of CAMITAX, we measured the resources consumed by the Docker containers run by Nextflow, during the analysis run, which are responsible for the analysis.

## Results

The kMetaShot reference was built on 253 628 bacterial and 1289 archaeal RefSeq genomes downloaded on 31 July 2022 and corresponding to 64 320 strains (according to the kMetaShot refinement system), 37 639 species, and 3713 genera.

### Testing on Human Microbiome Project genomes and benchmarking

**Figure 4:**
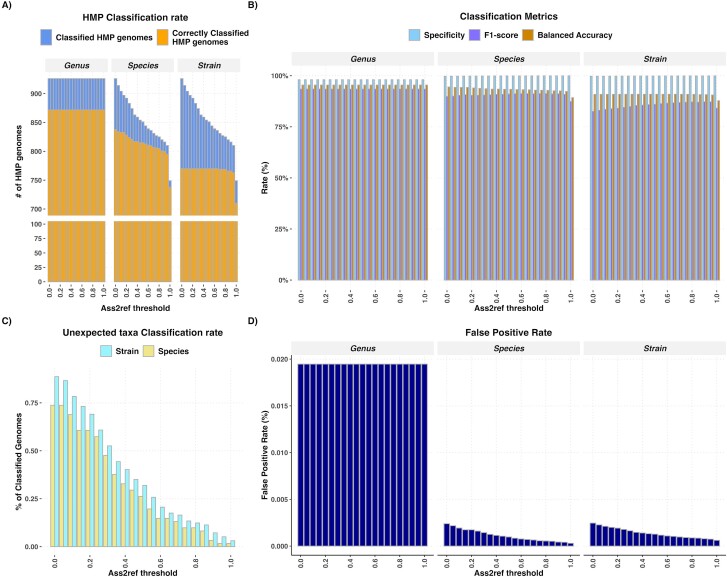
kMetaShot classification performances on HMP genomes according to different ass2ref thresholds and stratified per taxonomic rank (i.e. strain, species, and genus levels). (A) Stacked barplot representing the number of assigned and correctly assigned genomes. (B) Barplot representing the observed specificity, F1-score, and BA according to ass2ref. (C) Number of species and strains not represented in the kMetaShot reference and not correctly classified. (D) Barplot representing the observed FPR according to increasing ass2ref and stratified per taxonomic rank.

kMetaShot was used to classify the HMP genomes to evaluate the reliability of its classification strategy. The 939 (937 bacteria and 2 archaea) HMP genomes span 190 genera, 556 species, and 934 strains and were selected considering 97 strains, 61 species, and 10 genera were not represented in the kMetaShot reference taxonomy being not included in RefSeq. kMetaShot was able to classify 926 HMP genomes (Fig. 4A), and the obtained results were compared with the expected strains, species, and genera. It correctly assigned 770, 830, and 872 genomes at strain, species, and genus levels, respectively, with an inferred sensitivity of around 82.0%, 89.2%, and 92.9% at strain, species, and genus levels, respectively. BA ranged between 90.8% and 95.4% (Fig. 4B and Supplementary Table 2). Considering the FPR, we observed values ranging from 0.2% (strains and species) to 1.9% (genus).

Successively, we focused on Fp and especially on taxa that are not included in the kMetaShot reference taxonomy (Fig. 4C). We found that only 11 strains and 2 species were not classified at all. This result highlighted the need to implement a threshold to improve classification accuracy. In particular, we implemented ass2ref (the ratio among the observed and expected taxa minimizer), as a measure of the reliability of the kMetaShot classifications. We tuned the ass2ref threshold from 0 to 1 observing a contraction of FPR and an improvement of F1-score at strain and genus levels (Fig. 4B and Supplementary Table 3). BA trends at species and strain levels were inversely proportional to ass2ref, due to a decrease in sensitivity. Furthermore, at the strain level, we observed that the number of correctly classified MAGs does not change for ass2ref values up to 0.65, demonstrating the robustness of the classification approach (Supplementary Table 3). On the contrary, the overall number of classified MAGs decreased, reducing the FPR. At the species level, ass2ref impacts both the number of classified and correctly classified MAGs. However, up to ass2ref equal to 0.15 the number of correctly classified MAGs remained partially affected (from 838 to 833) while the overall classification rate decreased from 926 to 897. Considering MAGs belonging to taxa not represented in the kMetaShot reference, the number of assigned taxa at both species and strain levels decreased as ass2ref increased (Fig. 4C). At species level a first leap is observed at ass2ref equal to 0.15, with the number of genomes not represented in kMetaShot reference decreasing from 45 to 37 (Supplementary Table 3). Regarding strain rank, with ass2ref set to 0.25, the genomes unexpectedly classified decreased from 86 to 59 (Supplementary Table 3). Taking together all these data and defining a threshold minimizing FPR but not penalizing sensitivity, we suggest setting up 0.2 as the ass2ref threshold.

We then re-evaluated the classification performances by using the 0.2 ass2ref threshold and observed an improvement in F1-measure at strain (84.11%), species (90.44%), and genus levels (93.51%). The balanced accuracy was not affected (Supplementary Table 4). HMP genomes were also classified using two ‘state-of-the-art’ tools, namely, CAMITAX and GTDBtk, and their performances were compared with kMetaShot. Both CAMITAX and GTDBtk reached a sensitivity and BA lower than kMetaShot at both species and genus levels (Supplementary Table 4), while they are not able to provide classifications at the strain level.

### Benchmarking on the *in silico* Critical Assessment of Metagenome Interpretation II datasets

**Figure 5:**
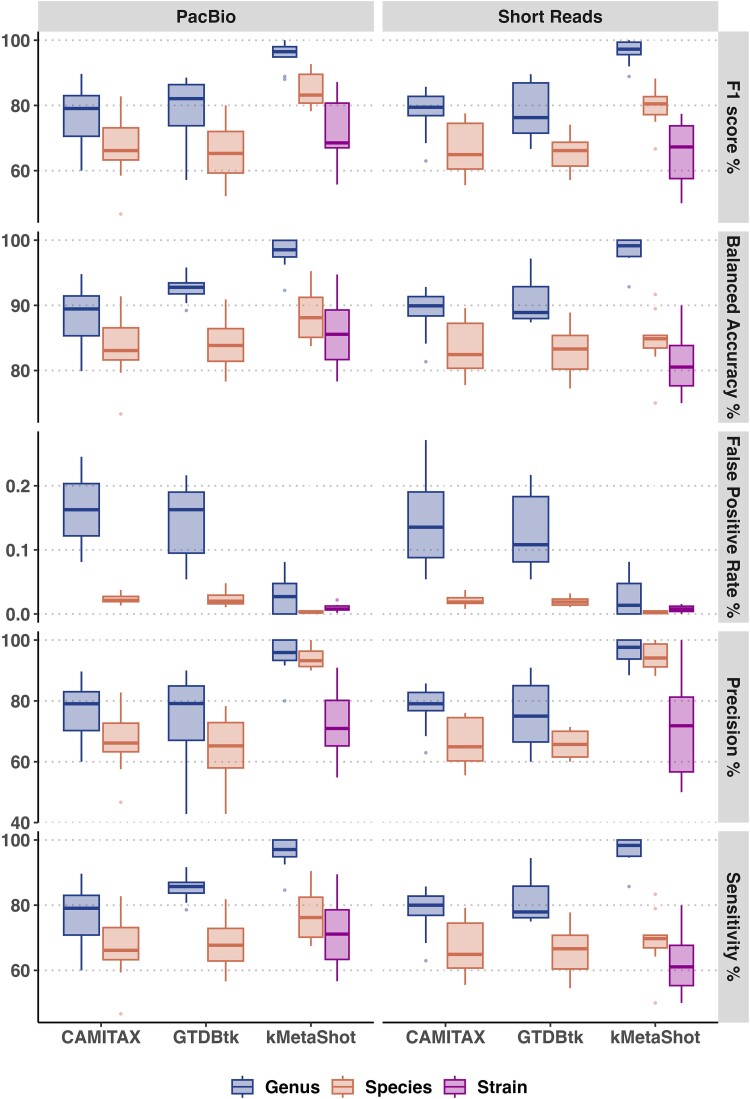
Boxplots representing the sensitivity, precision, FPR, BA, and F1-score distribution measured for the benchmarked tools for both PacBio- and Illumina-simulated reads in the CAMI2 GI data. Data are stratified according to the analysed taxonomic rank (i.e. genus, species, and strain).

The selected tools were also benchmarked using datasets provided by the CAMI II initiative. For each dataset, available contigs were binned and the obtained MAGs were classified. For kMetaShot, an ass2ref of 0.2 was set for all datasets. The measured sensitivity, precision, balanced accuracy, F1-score, and FPR are graphically represented as box plots in Fig. 5 and Supplementary Figs 1–5. kMetaShot obtained the highest sensitivity at species and genus levels in GI-Illumina (median 69.75 IQR 3.86, median 98.33 IQR 4.989) and GI-PacBio (median 76.20 IQR 12.26, median 97.07 IQR 5.13) (Fig. 5), in Air-Illumina at the genus level (median 100 IQR 0), and Air-PacBio at the genus level (median 98.83 IQR 1.94). kMetaShot achieved the best precision score for all datasets at species and genus levels (Fig. 5 and Supplementary Fig. 2). The BA performances of kMetaShot were higher than those of other tools at the genus level for all data sets and at the species level for GI-PacBio and GI-Illumina (Fig. 5 and Supplementary Fig. 3). The BA of kMetaShot and CAMITAX was comparable for Air-Illumina (median 72.79 versus 75.59 and IQR 12.66 versus 13.57) as reported in Supplementary Fig. 3. Lastly, the F1-score was higher for kMetaShot in all datasets at genus and species levels and comparable with CAMITAX at the species level for Air-Illumina (median 84.23 versus 84.96, IQR 10.10 versus 13.13, Fig. 5 and Supplementary Fig. 4).

### Benchmarking on real sequencing data

kMetaShot was also benchmarked by using real mock data by Shakya *et al*. [60]. This dataset was generated by sequencing a community made up of 48 bacterial and 16 archaeal genomes (accounting for 62 species and 50 genera, Supplementary Table 1). We performed the meta-assembly by using the two most cited and widely employed tools, namely, MegaHIT and metaSPAdes. 65 and 63 MAGs were obtained with metaSPAdes and MegaHIT, respectively. These MAGs were taxonomically classified with kMetaShot, CAMITAX, and GTDBtk, and the results were compared against the expected strains, species, and genera (Table 2 and Table 3). The custom ass2ref filter was set up to 0.2.

**Table 2:** Classification performance metrics measured on the real mock sequencing data.

	**Strain**		**Species**			**Genus**		
	**kMS**	**kMS corr.**	**kMS**	**Gtk**	**CMX**	**kMS**	**Gtk**	**CMX**
**tot MAGs**	63	63	63	63	63	63	63	63
**assigned MAGs**	49	49	49	58	58	59	59	60
**corr. Ass. MAGs**	9	9 + 33	49	54	55	59	55	58
**Sensitivity %**	52.94	83.67	75.81	82.26	80.65	92.00	88.00	92.00
**FPR %**	0.00	0.01	0.00	0.00	0.00	0.00	0.00	0.00
**Precision %**	19.15	87.23	100.00	92.73	96.15	100.00	91.67	97.87
**BA %**	76.44	91.84	87.90	91.12	90.32	96.00	93.95	95.99
**F1-score %**	28.13	85.42	86.24	87.18	87.72	95.83	89.80	94.85
**Tp**	9	9 + 32	47	51	50	46	44	46
**Tn**	64,334	64,334	37,645	37,641	37,643	3664	3660	3663
**Fp**	38	6	0	4	2	0	4	1
**Fn**	8	8	15	11	12	4	6	4

MAGs obtained through the binning of MegaHIT contigs were classified by kMetaShot (kMS), GTDBtk (Gtk), and CAMITAX (CMX) at strain (St, for kMS only), species (Sp), and genus (G) levels. kMS corr. correspond to the refined evaluation of kMetaShot classification following the repositioning of genomes lacking a strain classification (abbreviations: correctly assigned MAGs is corr. ass. MAGs; balanced accuracy is BA; kMetaShot corrected is kMS corr.).

The results shown in Table 2 and Table 3 demonstrated that the classification performances were meta-assembler independent for all the tested tools. In addition, kMetaShot correctly assigned the highest number of MAGs with the lowest number of Fp at both species and genus levels (Table 2 and Table 3). Considering 47 out 64 mock genomes lack a classification at the strain level making kMetaShot performances evaluation biased [60], we further evaluated the subset of MAGs assigned at the strain level (38 and 39 for MegaHIT and MetaSpades, respectively), by measuring the ANI index against the RefSeq genomes available for all the observed strains. The result of the pairwise comparison pointed out the kMetaShot classification (Supplementary Figs 6 and 7) is supported by the evidence of an ANI index higher than 97%. Accordingly, the number of correctly assigned MAGs at the strain level was 42 and 43 for metaSpades (Table 3) and MegaHIT (Table 2) MAGs, respectively, increasing both precision and BA. Notably, both GTDBtk and CAMITAX obtained a slightly higher BA at the species level and CAMITAX at the genus level as a result of the higher sensitivity on MetaSPAdes MAGs. Nonetheless, kMetaShot is the most precise tool in all the taxonomic levels with both meta-assemblers MAG sets.

**Table 3:** Classification performance metrics measured on the real mock sequencing data.

	**Strain**		**Species**			**Genus**		
	**kMS**	**kMS corr.**	**kMS**	**Gtk**	**CMX**	**kMS**	**Gtk**	**CMX**
**tot MAGs**	65	65	65	65	65	65	65	65
**assigned MAGs**	49	49	49	59	58	60	59	60
**corr. ass. MAGs**	8	8 + 35	49	55	55	60	55	58
**Sensitivity %**	41.18	80.00	74.19	80.65	80.65	90.00	86.00	92.00
**FPR %**	0.00	0.01	0.00	0.00	0.00	0.00	0.00	0.00
**Precision %**	15.22	86.96	100.00	92.59	96.15	100.00	91.49	97.87
**BA %**	70.56	83.48	87.10	90.32	90.32	95.00	92.95	95.99
**F1-score %**	22.22	83.33	85.19	86.21	87.72	94.74	88.66	94.85
**Tp**	7	7 + 33	46	50	50	45	43	46
**Tn**	64 333	64 333	37 645	37 641	37 643	3664	3660	3663
**Fp**	39	6	0	4	2	0	4	1
**Fn**	10	10	16	12	12	5	7	4

MAGs obtained through the binning of metaSpades contigs were classified by kMetaShot (kMS), GTDBtk (Gtk), and CAMITAX (CMX) at strain (St, for kMS only), species (Sp), and genus (G) levels. kMS corr. correspond to the refined evaluation of kMetaShot classification following the repositioning of genomes lacking a strain classification (abbreviations: correctly assigned MAGs is corr. ass. MAGs; balanced accuracy is BA; kMetaShot corrected is kMS corr.).

### Computational efficiency assessment

**Figure 6:**
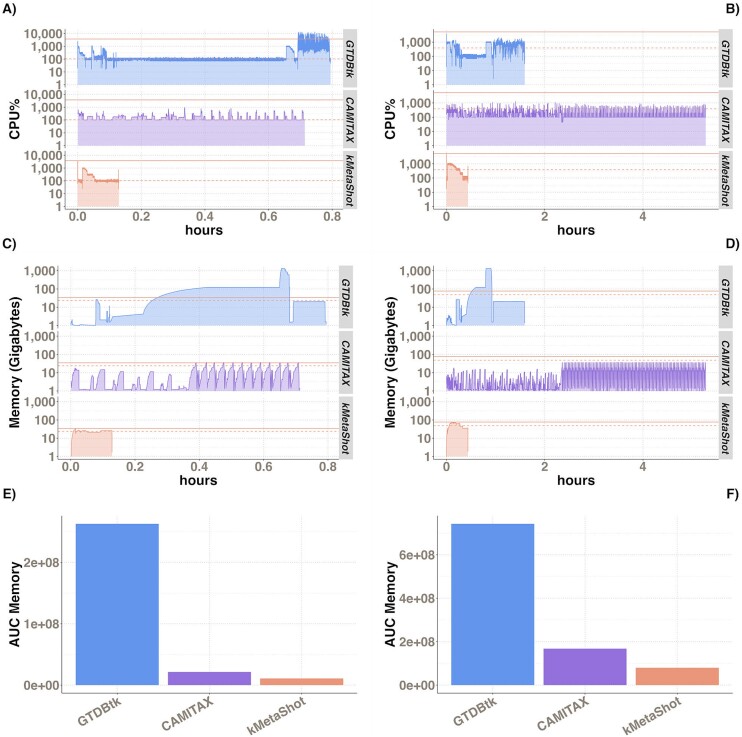
Evaluation of the computational resources required to address the classification task. In particular, RAM and CPU consumption were measured in hours. All tools were tested by using 10 CPUs. In (A), (C), and (E), the CPU load, memory engagement, and AUC for memory usage are shown, respectively, for Air-Illumina Sample12. (B), (D), and (F) show the same data for Air-PacBio Sample4.

The measurement of memory and CPU% usage with job runtime was carried out for the smallest and the largest samples in terms of MAG number, corresponding to Sample 12 in Air-Illumina and Sample 4 in Air-PacBio with 12 and 103 MAGs, respectively. For all tools, the default settings were used except for the CPU number, which was settled to 10. Regardless of the analysed datasets, the kMetaShot job runtimes were the shortest (Fig. 6). kMetaShot has the lowest CPU% usage among other tool curves. With respect to memory use, kMetaShot is second only to CAMITAX but with the shortest loading time (Fig. 6).

## Conclusions

Here, we present kMetaShot, a fast and reliable tool for performing MAG taxonomic classification. Furthermore, we provided evidence of its reliability in MAG classification regardless of the employed sequencing technology. In general, it outperformed GTDBtk and CAMITAX in most of the benchmarks considered using both simulated and real data. Moreover, its alignment-free design not requiring reference genomes and/or collections allows to significantly reduce the processing time, representing a valid alternative to the benchmarked tools. Furthermore, considering computational resources consumption kMetaShot is particularly effective compared to GTDBtk, the most commonly used tool for MAG taxonomic classification. It depends on the different approaches implemented by the two tools. Indeed, while kMetaShot is a stand-alone tool strictly requiring reference data, MAGs sequences, and ass2ref parameter setting, GTDBtk exploits a four-step workflow relying on multiple parameters and third-party tools. Nonetheless, the GTDB taxonomy project has become a standard in metagenomics because it attempts to resolve NCBI taxonomy inconsistencies in a phylogeny-oriented way. The advantages of using kMetaShot rely on its accuracy and short processing time compared to GTDBtk. On the other hand, phylogeny-based classification becomes particularly effective for MAGs lacking valuable references in public repositories. CAMITAX’s overall CPU usage is the lowest among the compared tools. Indeed, CAMITAX also performs a complex workflow requiring third-party tools, but its implementation relies on Nextflow, which allows it to efficiently exploit available resources. Nonetheless, in terms of maximum memory usage, CAMITAX and kMetaShot are comparable. Finally, it is worthy to note that the kMetaShot peaks in memory and CPU correspond to the reference loading in memory, requiring at least 32Gb. Nonetheless, the shortest execution time ensures to rapidly free resources.

Remarkably, kMetaShot has the unique feature of achieving taxonomic classification up to the strain/subspecies level. Finally, kMetaShot design and implementation made it an easy-to-install, through the CONDA package and environment manager, and easy-to-use tool, directly working on the binning output because for both task tools, a single operation is needed with few settleable parameters to choose.

**Key Points**

- Assembly-based shotgun metagenomics allows us to address the challenging task of identifying new genomes, genes, and pathways.
- Existing tools for MAG taxonomic classification are unable to classify at the strain level and are computationally/time expensive.
- kMetaShot is a reliable and computationally effective tool for MAG taxonomic classification up to the strain level.

## Supplementary Material

Supplementary_Figure_1_bbae680

Supplementary_Figure_2_bbae680

Supplementary_Figure_3_bbae680

Supplementary_Figure_4_bbae680

Supplementary_Figure_5_bbae680

Supplementary_Figure_6_bbae680

Supplementary_Figure_7_bbae680

Supplementary_Table_1_bbae680

Supplementary_Table_2_bbae680

Supplementary_Table_3_bbae680

Supplementary_Table_4_bbae680

## Data Availability

kMetaShot has been developed and tested on an HP Proliant server with 80 CPUs (160 threads), 2Tb RAM, and a CentOS 7 operative system. It is written in Python 3.7 and is available at https://github.com/gdefazio/kMetaShot and is distributed as the Conda package and Docker container. We suggest using kMetaShot on a machine having as minimum resources four CPUs and 32 Gb RAM. A periodic update of the kMetaShot reference will be carried out every year and will be distributed via GitHub. Benchmark scripts are also available on GitHub, and related data are published at https://zenodo.org/doi/10.5281/zenodo.11122221. The scripts used for evaluating CAMITAX performance and the corresponding documentation are available on GitHub (https://github.com/mtangaro/camitax-usage-eval).

## References

[1] Barton L , Northup DE. *Microb Ecol* Wiley‐Blackwell, 2011. 10.1002/9781118015841.

[2] Berg G , Rybakova D, Fischer D. et al. Microbiome definition re-visited: Old concepts and new challenges. *Microbiome* 2020;8:103. 10.1186/s40168-020-00875-0. 32605663 PMC7329523

[3] Blevins SM , Bronze MS. Robert Koch and the ‘golden age’ of bacteriology. *Int J Infect Dis* 2010;14:e744–51. 10.1016/j.ijid.2009.12.003. 20413340

[4] Bassler BL . Small talk: Cell-to-cell communication in bacteria. *Cell* 2002;109:421–4. 10.1016/S0092-8674(02)00749-3. 12086599

[5] Metchnikoff E. The prolongation of life: optimistic studies. https://www.gutenberg.org/files/51521/51521-h/51521-h.htm.

[6] Leimbach A , Hacker J, Dobrindt UE. E. coli as an all-rounder: The thin line between commensalism and pathogenicity. In: Between Pathogenicity and Commensalism. Springer, 2013;358:3–32. 10.1007/82_2012_303. 23340801

[7] Koch H , Germscheid N, Freese HM. et al. Genomic, metabolic and phenotypic variability shapes ecological differentiation and intraspecies interactions of Alteromonas macleodii. *Sci Rep* 2020;10:809. 10.1038/s41598-020-57526-5. 31964928 PMC6972757

[8] Van Rossum T , Ferretti P, Maistrenko OM. et al. Diversity within species: Interpreting strains in microbiomes. *Nat Rev Microbiol* 2020;18:491–506. 10.1038/s41579-020-0368-1. 32499497 PMC7610499

[9] Richter M , Rosselló-Móra R. Shifting the genomic gold standard for the prokaryotic species definition. *Proc Natl Acad Sci* 2009;106:19126–31. 10.1073/pnas.0906412106. 19855009 PMC2776425

[10] Mende DR , Sunagawa S, Zeller G. et al. Accurate and universal delineation of prokaryotic species. *Nat Methods* 2013;10:881–4. 10.1038/nmeth.2575. 23892899

[11] Bikel S , Valdez-Lara A, Cornejo-Granados F. et al. Combining metagenomics, metatranscriptomics and viromics to explore novel microbial interactions: Towards a systems-level understanding of human microbiome. *Comput Struct Biotechnol J* 2015;13:390–401. 10.1016/j.csbj.2015.06.001. 26137199 PMC4484546

[12] Segata N . On the road to strain-resolved comparative metagenomics. *mSystems* 2018;3. 10.1128/mSystems.00190-17. PMC5850074 29556534

[13] Ercolini D . High-throughput sequencing and metagenomics: Moving forward in the culture-independent analysis of food microbial ecology. *Appl Environ Microbiol* 2013;79:3148–55. 10.1128/AEM.00256-13. 23475615 PMC3685257

[14] Mapelli F , Scoma A, Michoud G. et al. Biotechnologies for marine oil spill cleanup: Indissoluble ties with microorganisms. *Trends Biotechnol* 2017;35:860–70. 10.1016/j.tibtech.2017.04.003. 28511936

[15] Ullah S , He P, Ai C. et al. How do soil bacterial diversity and community composition respond under recommended and conventional nitrogen fertilization regimes? *Microorganisms* 2020;8:1193. 10.3390/microorganisms8081193. 32764443 PMC7466009

[16] Nayfach S , Roux S, Seshadri R. et al. A genomic catalog of Earth’s microbiomes. *Nat Biotechnol* 2021;39:499–509. 10.1038/s41587-020-0718-6. 33169036 PMC8041624

[17] Lozupone CA , Stombaugh JI, Gordon JI. et al. Diversity, stability and resilience of the human gut microbiota. *Nature* 2012;489:220–30. 22972295 10.1038/nature11550 PMC3577372

[18] Blanco-Míguez A , Beghini F, Cumbo F. et al. Extending and improving metagenomic taxonomic profiling with uncharacterized species using MetaPhlAn 4. *Nat Biotechnol* 2023;41:1633–44. 10.1038/s41587-023-01688-w. PMC10635831 36823356

[19] Wood DE , Salzberg SL. Kraken: Ultrafast metagenomic sequence classification using exact alignments. *Genome Biol* 2014;15:R46. 10.1186/gb-2014-15-3-r46. 24580807 PMC4053813

[20] Wood DE , Lu J, Langmead B. Improved metagenomic analysis with kraken 2. *Genome Biol* 2019;20:257. 10.1186/s13059-019-1891-0. 31779668 PMC6883579

[21] Fosso B , Santamaria M, D’Antonio M. et al. MetaShot: An accurate workflow for taxon classification of host-associated microbiome from shotgun metagenomic data. *Bioinformatics* 2017;33:1730–2. 10.1093/bioinformatics/btx036. 28130230 PMC5447231

[22] Dotan E , Alburquerque M, Wygoda E. et al. GenomeFLTR: Filtering reads made easy. *Nucleic Acids Res* 2023;51:W232–6. 10.1093/nar/gkad410. 37177997 PMC10320065

[23] Zhu K , Schäffer AA, Robinson W. et al. Strain level microbial detection and quantification with applications to single cell metagenomics. *Nat Commun* 2022;13:6430. 10.1038/s41467-022-33869-7. 36307411 PMC9616933

[24] Raju RS , Al Nahid A, Chondrow DP. et al. VirusTaxo: Taxonomic classification of viruses from the genome sequence using k-mer enrichment. *Genomics* 2022;114:110414. 10.1016/j.ygeno.2022.110414. 35718090

[25] Bowers RM , Kyrpides NC, Stepanauskas R. et al. Minimum information about a single amplified genome (MISAG) and a metagenome-assembled genome (MIMAG) of bacteria and archaea. *Nat Biotechnol* 2017;35:725–31. 10.1038/nbt.3893. 28787424 PMC6436528

[26] Dijkshoorn L , Ursing BM, Ursing JB. Strain, clone and species: Comments on three basic concepts of bacteriology. *J Med Microbiol* 2000;49:397–401. 10.1099/0022-1317-49-5-397. 10798550

[27] Hugenholtz P , Skarshewski A, Parks DH. Genome-based microbial taxonomy coming of age. *Cold Spring Harb Perspect Biol* 2016;8:1–11. 10.1101/cshperspect.a018085. PMC4888819 26988968

[28] Nayfach S , Rodriguez-Mueller B, Garud N. et al. An integrated metagenomics pipeline for strain profiling reveals novel patterns of bacterial transmission and biogeography. *Genome Res* 2016;26:1612–25. 10.1101/gr.201863.115. 27803195 PMC5088602

[29] Chang T , Gavelis GS, Brown JM. et al. Genomic representativeness and chimerism in large collections of SAGs and MAGs of marine prokaryoplankton. *Microbiome* 2024;12:126. 39010229 10.1186/s40168-024-01848-3 PMC11247762

[30] Whipps JM , Lewis K, Cooke R. Mycoparasitism and plant disease control. In: *Fungi in Biological Control Systems*. Manchester University Press, 1988;1988:161–87.

[31] Brenner K , You L, Arnold FH. Engineering microbial consortia: A new frontier in synthetic biology. *Trends Biotechnol* 2008;26:483–9. 10.1016/j.tibtech.2008.05.004. 18675483

[32] Teague BP , Weiss R. Synthetic communities, the sum of parts. *Science* 2015;349:924–5. 10.1126/science.aad0876. 26315419

[33] Neuenschwander SM , Ghai R, Pernthaler J. et al. Microdiversification in genome-streamlined ubiquitous freshwater actinobacteria. *ISME J* 2018;12:185–98. 10.1038/ismej.2017.156. 29027997 PMC5739012

[34] Nowrouzian FL , Adlerberth I, Wold AE. Enhanced persistence in the colonic microbiota of Escherichia coli strains belonging to phylogenetic group B2: Role of virulence factors and adherence to colonic cells. *Microbes Infect* 2006;8:834–40. 16483819 10.1016/j.micinf.2005.10.011

[35] Kang D , Li F, Kirton ES. et al. MetaBAT 2: An adaptive binning algorithm for robust and efficient genome reconstruction from metagenome assemblies. *PeerJ* 2019;7:e7359. 31388474 10.7717/peerj.7359 PMC6662567

[36] Wu Y-W , Simmons BA, Singer SW. MaxBin 2.0: An automated binning algorithm to recover genomes from multiple metagenomic datasets. *Bioinformatics* 2016;32:605–7. 10.1093/bioinformatics/btv638. 26515820

[37] Chaumeil P-A , Mussig AJ, Hugenholtz P. et al. GTDB-Tk: A toolkit to classify genomes with the genome taxonomy database. *Bioinformatics* 2020;36:1925–7. 10.1093/bioinformatics/btz848. PMC7703759 31730192

[38] Chaumeil P-A , Mussig AJ, Hugenholtz P. et al. GTDB-Tk v2: Memory friendly classification with the genome taxonomy database. *Bioinformatics* 2022;38:5315–6. 10.1093/bioinformatics/btac672. 36218463 PMC9710552

[39] Bremges A , Fritz A, McHardy AC.. McHardy AC. CAMITAX: Taxon labels for microbial genomes. GigaScience, 2020;9:9. 10.1093/gigascience/giz154. PMC6946028 31909794

[40] Patangia DV , Grimaud G, O’Shea C-A. et al. Early life exposure of infants to benzylpenicillin and gentamicin is associated with a persistent amplification of the gut resistome. *Microbiome* 2024;12:19. 10.1186/s40168-023-01732-6. 38310316 PMC10837951

[41] Meyer F , Fritz A, Deng Z-L. et al. Critical assessment of metagenome interpretation: The second round of challenges. *Nat Methods* 2022;19:429–40. 10.1038/s41592-022-01431-4. 35396482 PMC9007738

[42] Moeckel C , Mareboina M, Konnaris MA. et al. A survey of k-mer methods and applications in bioinformatics. *Comput Struct Biotechnol J* 2024;23:2289–303. 10.1016/j.csbj.2024.05.025. 38840832 PMC11152613

[43] Bankevich A , Nurk S, Antipov D. et al. SPAdes: A new genome assembly algorithm and its applications to single-cell sequencing. *J Comput Biol* 2012;19:455–77. 10.1089/cmb.2012.0021. 22506599 PMC3342519

[44] Nurk S , Meleshko D, Korobeynikov A. et al. metaSPAdes: A new versatile metagenomic assembler. *Genome Res* 2017;27:824–34. 10.1101/gr.213959.116. 28298430 PMC5411777

[45] Li D , Liu C-M, Luo R. et al. MEGAHIT: An ultra-fast single-node solution for large and complex metagenomics assembly via succinct de Bruijn graph. *Bioinformatics* 2015;31:1674–6. 10.1093/bioinformatics/btv033. 25609793

[46] Zerbino DR , Birney E. Velvet: Algorithms for de novo short read assembly using de Bruijn graphs. *Genome Res* 2008;18:821–9. 10.1101/gr.074492.107. 18349386 PMC2336801

[47] Namiki T , Hachiya T, Tanaka H. et al. MetaVelvet: An extension of velvet assembler to de novo metagenome assembly from short sequence reads. *Nucleic Acids Res* 2012;40:e155. 10.1093/nar/gks678. 22821567 PMC3488206

[48] Menzel P , Ng KL, Krogh A. Fast and sensitive taxonomic classification for metagenomics with kaiju. *Nat Commun* 2016;7:11257. 10.1038/ncomms11257. 27071849 PMC4833860

[49] Li H . Minimap2: Pairwise alignment for nucleotide sequences. *Bioinformatics* 2018;34:3094–100. 10.1093/bioinformatics/bty191. 29750242 PMC6137996

[50] Chor B , Horn D, Goldman N. et al. Genomic DNA k-mer spectra: Models and modalities. *Genome Biol* 2009;10:R108. 10.1186/gb-2009-10-10-r108. 19814784 PMC2784323

[51] Turnbaugh PJ , Ley RE, Hamady M. et al. The Human Microbiome Project. *Nature* 2007;449:804–10. 10.1038/nature06244. 17943116 PMC3709439

[52] O’Leary NA , Wright MW, Brister JR. et al. Reference sequence (RefSeq) database at NCBI: Current status, taxonomic expansion, and functional annotation. *Nucleic Acids Res* 2016;44:D733–45. 10.1093/nar/gkv1189. 26553804 PMC4702849

[53] Gil R , Latorre A. Factors behind junk DNA in bacteria. *Genes* 2012;3:634–50. 10.3390/genes3040634. 24705080 PMC3899985

[54] Ondov BD , Treangen TJ, Melsted P. et al. Mash: Fast genome and metagenome distance estimation using MinHash. *Genome Biol* 2016;17:132. 10.1186/s13059-016-0997-x. 27323842 PMC4915045

[55] Konstantinidis KT , Tiedje JM. Genomic insights that advance the species definition for prokaryotes. *Proc Natl Acad Sci USA* 2005;102:2567–72. 10.1073/pnas.0409727102. 15701695 PMC549018

[56] Roberts M , Hayes W, Hunt BR. et al. Reducing storage requirements for biological sequence comparison. *Bioinformatics* 2004;20:3363–9. 10.1093/bioinformatics/bth408. 15256412

[57] Senuma H , Hayashi K, Tsuzuki M. Contribution of the sensor histidine kinases PhcS and VsrA to the quorum sensing of Ralstonia pseudosolanacearum strain OE1-1. *Mol Plant-Microbe Interact* 2024, 2024;37:688–97. 10.1094/MPMI-05-24-0049-R. 39295141

[58] Babraham Bioinformatics - FastQC A Quality Control tool for High Throughput Sequence Data . 2018.

[59] Huang M , Li L, Wen S. et al. Hybridization chain reaction and magnetic beads-assisted highly sensitive detection of microRNA-21 with helical gold nanorods as dark-filed light scattering optical probe. *Talanta* 2024, 2024;285:127382. 10.1016/j.talanta.2024.127382. 39681057

[60] Shakya M , Quince C, Campbell JH. et al. Comparative metagenomic and rRNA microbial diversity characterization using archaeal and bacterial synthetic communities. *Environ Microbiol* 2013;15:1882–99. 10.1111/1462-2920.12086. 23387867 PMC3665634

[61] Yousefian M . GitHub - Manzik/Cmdbench: Quick and Easy Resource Usage Monitoring and Benchmarking for any command’s CPU, Memory, Disk Usage and Runtime. 2020. https://github.com/manzik/cmdbench.

