# Supplementary material for: kMetaShot: a fast and reliable taxonomy classifier for metagenome-assembled genomes
Source: Brief Bioinform. 2025 Jan 2;26(1):bbae680. doi: 10.1093/bib/bbae680 (PMC11695915; doi:10.1093/bib/bbae680)
Supplement: Supplementary_Figure_1_bbae680 [file supplementary_figure_1_bbae680.docx]

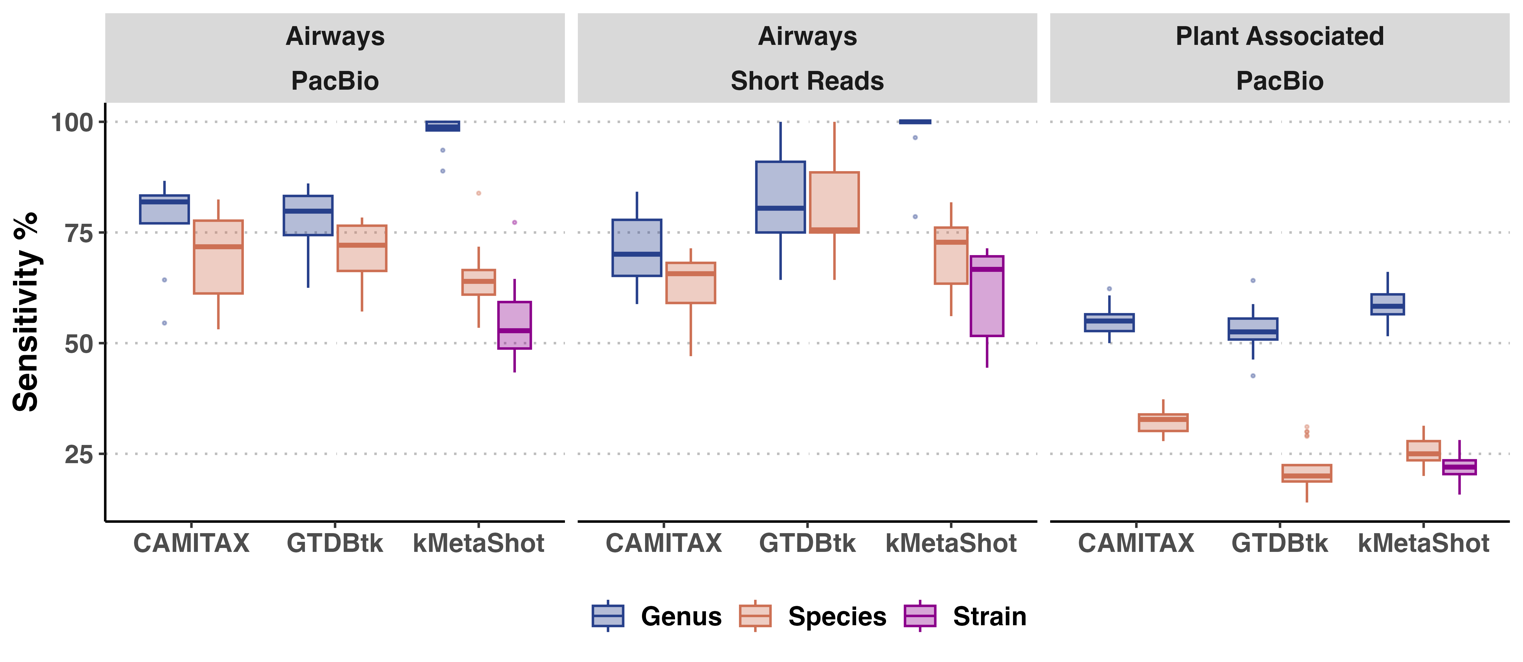
Supplementary Figure 1: Box plots representing inferred Sensitivity on CAMI II mock communities. Performances of compared tools are shown at Genus, Species and Strain levels.
