# Supplementary material for: kMetaShot: a fast and reliable taxonomy classifier for metagenome-assembled genomes
Source: Brief Bioinform. 2025 Jan 2;26(1):bbae680. doi: 10.1093/bib/bbae680 (PMC11695915; doi:10.1093/bib/bbae680)
Supplement: Supplementary_Figure_4_bbae680 [file supplementary_figure_4_bbae680.docx]

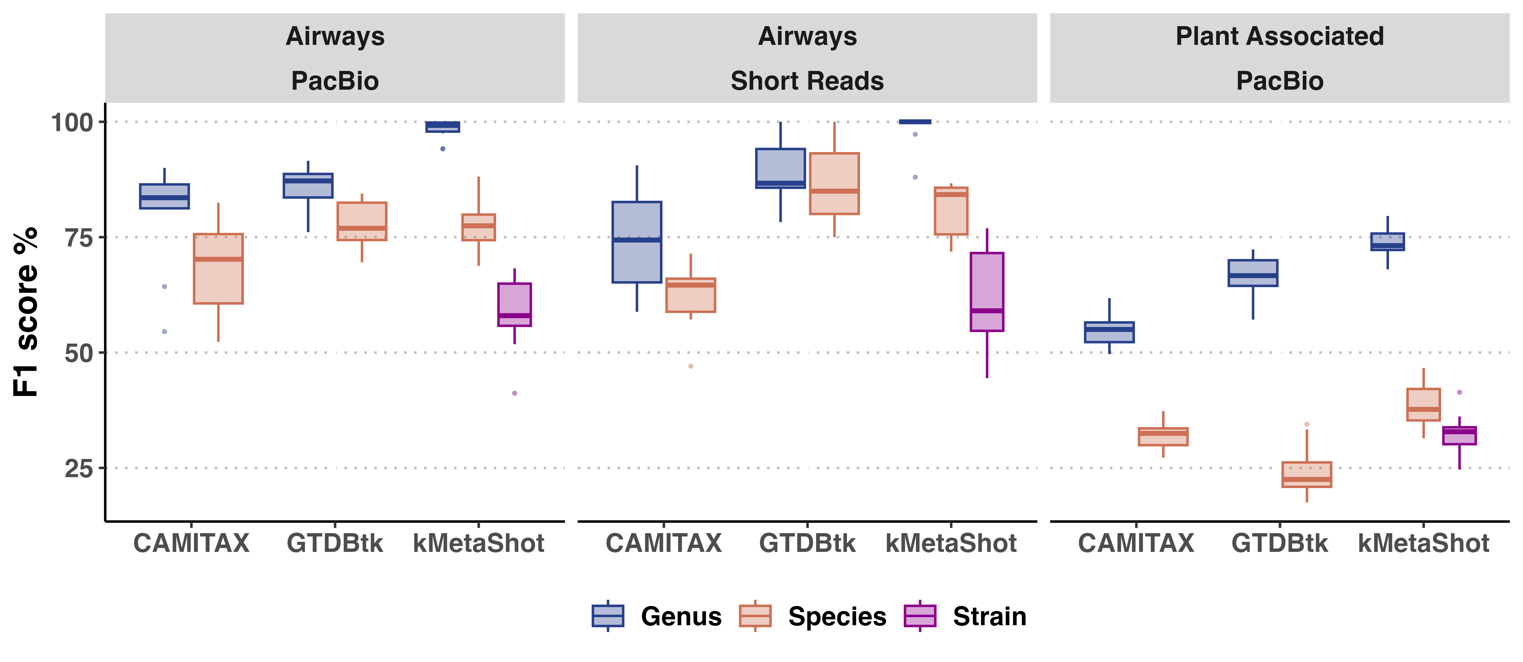
 Supplementary Figure 4: Box plots for F1 score of CAMI II mock communities. Performances of compared tools are visualized for Genus, Species and Strain level.
