# Supplementary material for: kMetaShot: a fast and reliable taxonomy classifier for metagenome-assembled genomes
Source: Brief Bioinform. 2025 Jan 2;26(1):bbae680. doi: 10.1093/bib/bbae680 (PMC11695915; doi:10.1093/bib/bbae680)
Supplement: Supplementary_Figure_6_bbae680 [file supplementary_figure_6_bbae680.docx]

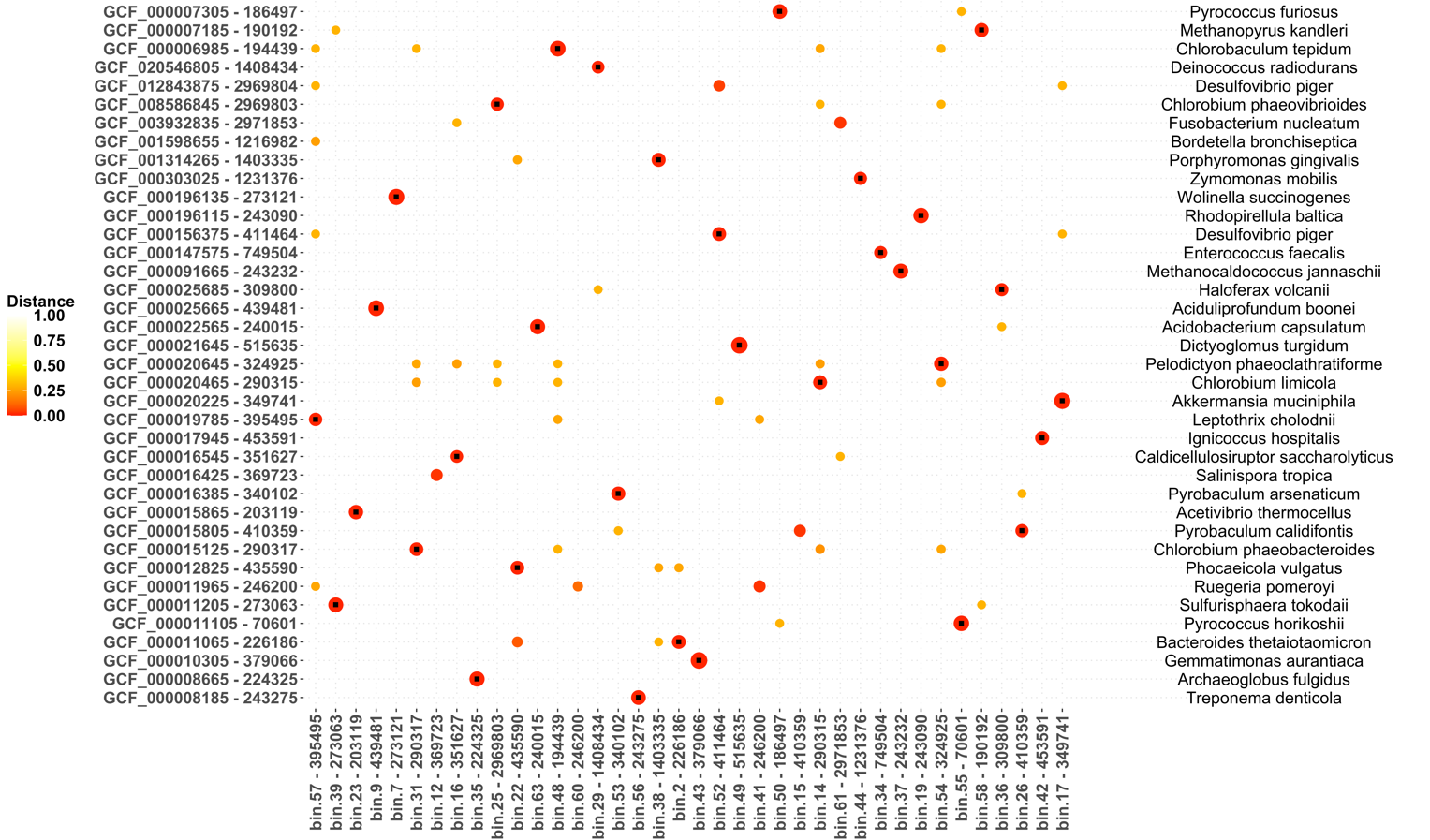


Supplementary Figure 6: HeatMap representing matrix obtained by inferring the ANI index through MASH and comparing MegaHIT MAGs lackcing a strain reference classification but with a kMetaShot classification at strain level (y axis) against RefSeq genome according to the observed kMetaShot classification (x axis). The x-axis labels report the RefSeq genome accession number and the NCBI classification taxid. The y-axis labels report the bin name and the kMetaShot classification expressed as taxid. For each MAG the lowest distance value represents the match with reference genome. The lower is the ANI distance the bigger is the dot size. The ANI ≥ 97% are indicated by a black point on the dot centre.
