# Supplementary material for: kMetaShot: a fast and reliable taxonomy classifier for metagenome-assembled genomes
Source: Brief Bioinform. 2025 Jan 2;26(1):bbae680. doi: 10.1093/bib/bbae680 (PMC11695915; doi:10.1093/bib/bbae680)
Supplement: Supplementary_Table_2_bbae680 [file supplementary_table_2_bbae680.docx]

Supplementary Table 2: Comparison between kMetaShot and expected taxonomic classification for HMP genomes before the application of ass2ref threshold.

|  | **tot MAGs** | **assigned** | **correctly assigned** | **Sensitivity**  **%** | **FPR%** | **Precision**  **%** | **BA**  **%** | **F1score**  **%** | **Tp** | **Tn** | **Fp** | **Fn** |
| --- | --- | --- | --- | --- | --- | --- | --- | --- | --- | --- | --- | --- |
| **Strain** | 939 | 926 | 770 | 82.00 | 0.25 | 83.15 | 90.88 | 82.57 | 770 | 63,225 | 156 | 169 |
| **Species** | 939 | 926 | 838 | 89.24 | 0.24 | 90.50 | 94.50 | 89.87 | 838 | 36,612 | 88 | 101 |
| **Genus** | 939 | 926 | 872 | 92.86 | 1.95 | 44.17 | 95.46 | 93.51 | 872 | 2,720 | 54 | 67 |
