# Supplementary material for: kMetaShot: a fast and reliable taxonomy classifier for metagenome-assembled genomes
Source: Brief Bioinform. 2025 Jan 2;26(1):bbae680. doi: 10.1093/bib/bbae680 (PMC11695915; doi:10.1093/bib/bbae680)
Supplement: Supplementary_Table_4_bbae680 [file supplementary_table_4_bbae680.docx]

Supplementary Table 4: Comparison between kMetaShot, GTDBtk, CAMITAX and expected taxonomic classification for HMP genomes. The kMetaShot performance shown in this table refers to the implementation of the ass2ref filter.

|  | **Strain** | **Species** | | | **Genus** | | |
| --- | --- | --- | --- | --- | --- | --- | --- |
|  | **kMetaShot** | **kMetaShot** | **GTDBtk** | **CAMITAX** | **kMetaShot** | **GTDBtk** | **CAMITAX** |
| **tot MAGs** | 939 | 939 | 939 | 939 | 939 | 939 | 939 |
| **assigned MAGs** | 892 | 892 | 842 | 748 | 926 | 933 | 757 |
| **correctly assigned MAGs** | 770 | 828 | 731 | 651 | 872 | 805 | 701 |
| **sensitivity%** | 82.00 | 88.71 | 77.85 | 69.33 | 92.86 | 85.73 | 74.65 |
| **FPR** | 0.19 | 0.17 | 0.30 | 0.26 | 1.95 | 4.6 | 8.27 |
| **precision%** | 86.32 | 92.83 | 86.82 | 87.03 | 94.17 | 86.28 | 92.60 |
| **BA %** | 90.90 | 94.00 | 88.77 | 84.53 | 95.46 | 87.79 | 92.08 |
| **F1-score** | 84.11 | 90.44 | 82.09 | 77.18 | 93.51 | 86.00 | 82.66 |
| **Tp** | 770 | 828 | 731 | 651 | 872 | 805 | 701 |
| **Tn** | 63,259 | 36,636 | 36,589 | 36,603 | 2,720 | 2,646 | 2,718 |
| **Fp** | 122 | 64 | 111 | 97 | 54 | 128 | 56 |
| **Fn** | 169 | 115 | 208 | 288 | 67 | 134 | 238 |
