# Supplementary material for: kMetaShot: a fast and reliable taxonomy classifier for metagenome-assembled genomes
Source: Brief Bioinform. 2025 Jan 2;26(1):bbae680. doi: 10.1093/bib/bbae680 (PMC11695915; doi:10.1093/bib/bbae680)
Supplement: Supplementary file 11 [file supplementary_table_1_bbae680.docx]

Supplementary Table 1: Composition of the mock community published by Shakya et al., 2013 (SRR6[illegible]
composed by 48 Bacteria and 16 Archaea

Species	Strain	Phylum	Kingdom
Ignicoccus hospitalis			
Pyrobaculum aerophilum IM2	**IM2**		
Pyrobaculum arsenaticum		*Crenarchaeota*	
Pyrobaculum calidifontis			
Sulfolobus tokodaii			
Pyrococcus furiosus			
Pyrococcus horikoshii			
Aciduliprofundum boonei			***Archaea***
Archaeoglobus fulgidus			
Haloferax volcanii		*Euryarchaeota*	
Methanopyrus kandleri			
Methanosarcina acetivorans C2A	**C2A**		
Methanocaldococcus jannaschii			
Methanococcus maripaludis C5	**C5**		
Methanococcus maripaludis S2	**S2**		
Nanoarchaeum equitans		*Nanoarchaeota*	
Hydrogenobaculum sp. Y04AAS1			***Bacteria***
Persephonella marina EX-H1	**EX-H1**	*Aquificae*	
Sulfurihydrogenibium sp. YO3AOP1			
Sulfurihydrogenibium yellowstonense SS-5	**SS-5**		
Thermotoga neapolitana DSM 4359	**DSM 4359**		
Thermotoga petrophila RKU-1	**RKU-1**	*Thermotogae*	
Thermotoga sp. RQ2			
Deinococcus radiodurans R1	**R1**	*DeinococcI*	
Thermus thermophilus HB8	**HB8**	*ThermI*	
Dictyoglomus turgidum		*Dictyoglomi*	
Salinispora arenicola		*Actinobacteria*	
Salinispora tropica			
Chloroflexus aurantiacus J-10-fl	**J-10-fl**	*Chloroflexi*	
Herpetosiphon aurantiacus			
Nostoc sp. PCC 7120		*Cyanobacteria*	
Bacteroides thetaiotaomicron			
Bacteroides vulgatus		*Bacteroidetes*	
